# The Diagnosis and Treatment of Rasmussen’s Encephalitis: A Case Report

**DOI:** 10.7759/cureus.34075

**Published:** 2023-01-23

**Authors:** Swaragandha S Jadhav, Avinash P Dhok, Kajal R Mitra, Ashish N Ambhore

**Affiliations:** 1 Department of Radiodiagnosis, NKP Salve Institute of Medical Sciences and Research Centre, Nagpur, IND

**Keywords:** case report, autoimmune disease, neurological disorder, seizures, rasmussen’s encephalitis

## Abstract

Rasmussen’s encephalitis is a very rare type of chronic inflammatory disease of the brain. We report a case of a nine-year-old male patient who presented with seizures and cognitive impairment for six years. An MRI of the brain revealed significant cerebral hemiatrophy. The patient was on immunoglobulin therapy. We also engage in a review of the existing literature on Rasmussen’s encephalitis.

## Introduction

Rasmussen’s encephalitis is a rare neurological disorder. It usually affects only one hemisphere of the brain, and the involvement of both hemispheres is extremely rare. It occurs in the adolescent age group and presents as an autoimmune disease. It is a gradually progressive disease that usually manifests with refractory seizures and cognitive impairment. It is diagnosed by MRI of the brain and the treatment involves corticosteroids, immunoglobulins, or surgery.

## Case presentation

A nine-year-old male, born out of a non-consanguineous marriage, presented with complaints of flexion deformity of the right hand and intermittent episodes of generalized status epilepticus for six years. The patient was on treatment for seizures under a local practitioner and had never undergone an MRI brain before. He had a history of poor scholastic performance, but there was no history of fever or trauma. On general examination, the patient was conscious and well-oriented. He was able to count objects, state his full name, ride a tricycle, and draw a circle. As per the clinical examination, he had achieved the milestones by the age of three years, suggestive of delayed milestones. The patient was vitally stable and systemic examination was within normal limits. Immunization was up to date. The patient’s mother had not undergone ultrasound scans during her ANC period. She neither had a history of fever during pregnancy, nor a history of miscarriage. The patient had been born full-term by normal vaginal delivery and weighed 3 kg at the time of birth. He had immediately cried after birth.

**Figure 1:**
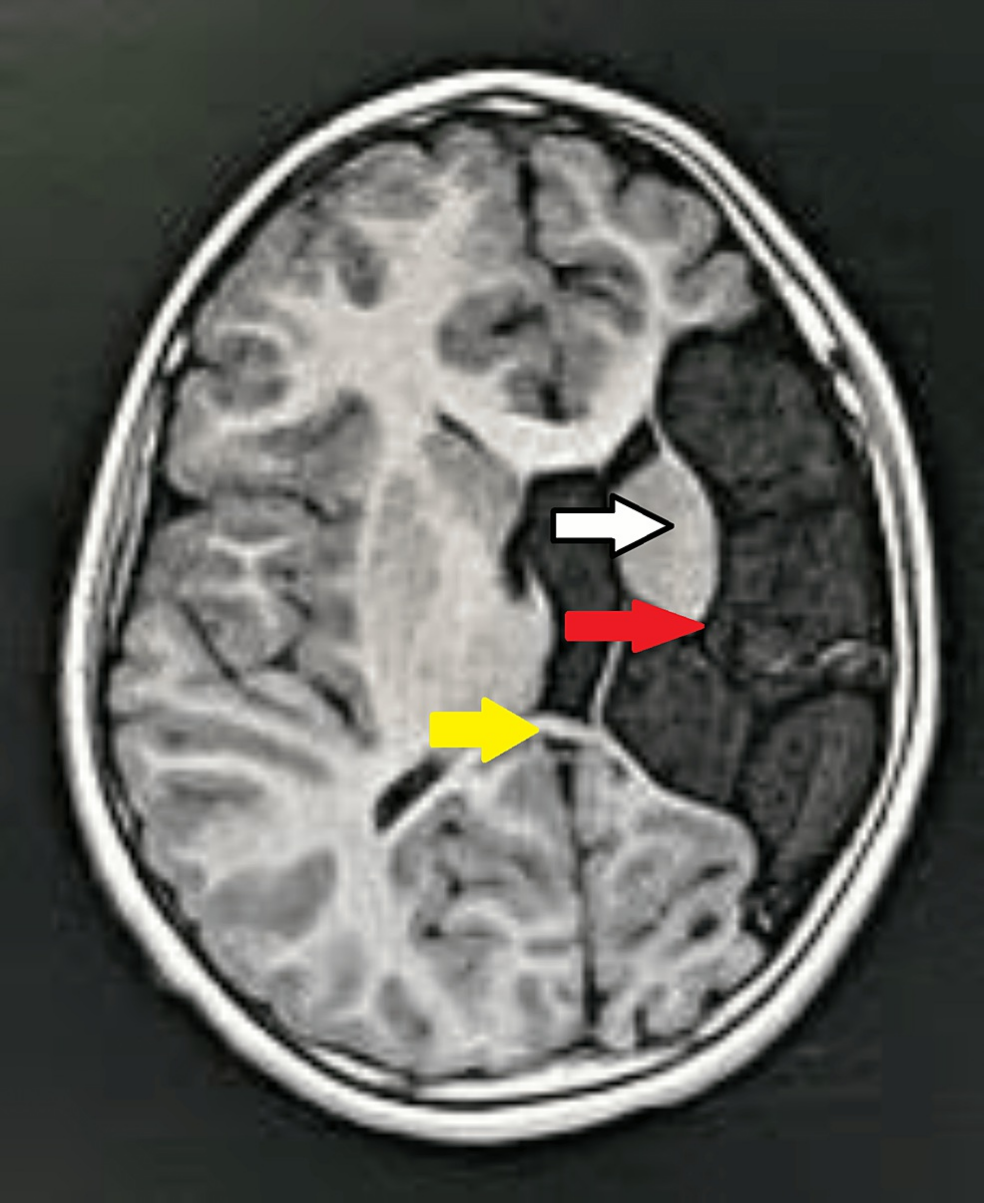
MRI brain axial T1WI Altered signal intensity is noted at the frontoparietal lobe on the left side with the loss of gray-white matter junction, encephalomalacia, and gliotic changes with dilatation of anterior horn and body of ipsilateral lateral ventricle appearing hypointense on T1WI suggestive of significant left-sided partial cerebral hemiatrophy. Atrophy of the splenium of the corpus callosum, lentiform nucleus, basal ganglia, and internal capsule on the left side is noted. Red arrow: altered signal intensity with encephalomalacia, and gliotic changes at the left frontoparietal lobe. White arrow: atrophic left gangliocapsular region. Yellow arrow: atrophic splenium MRI: magnetic resonance imaging; T1WI: T1 weighted imaging

**Figure 2:**
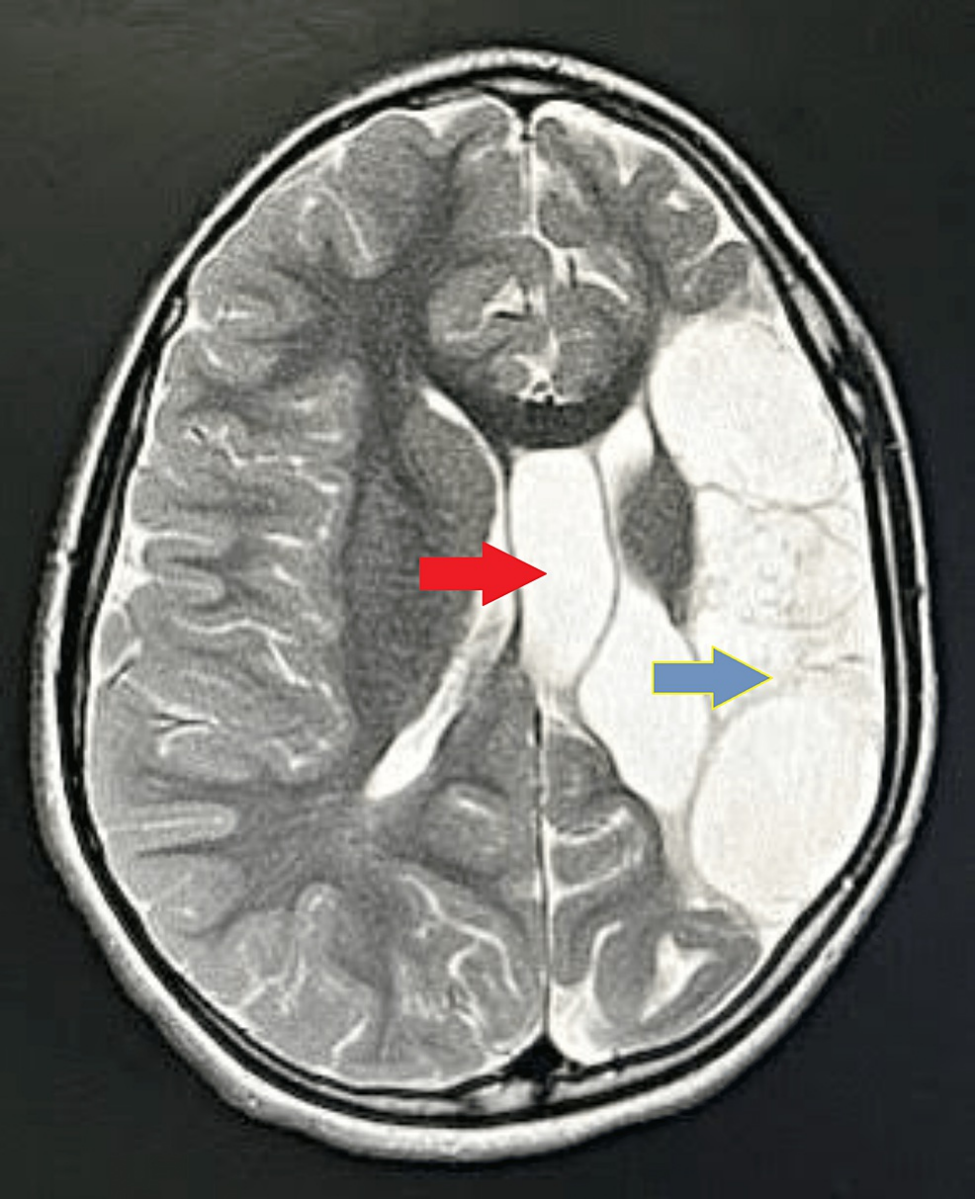
MRI brain axial T2WI Altered signal intensity is noted at the frontoparietal lobe on the left side with the loss of gray-white matter junction, encephalomalacia, and gliotic changes with dilatation of the anterior horn and body of ipsilateral lateral ventricle appearing hyperintense on T2WI Red arrow: dilated left lateral ventricle. Blue arrow: altered signal intensity at the left frontoparietal lobe MRI: magnetic resonance imaging; T2WI: T2 weighted imaging

**Figure 3:**
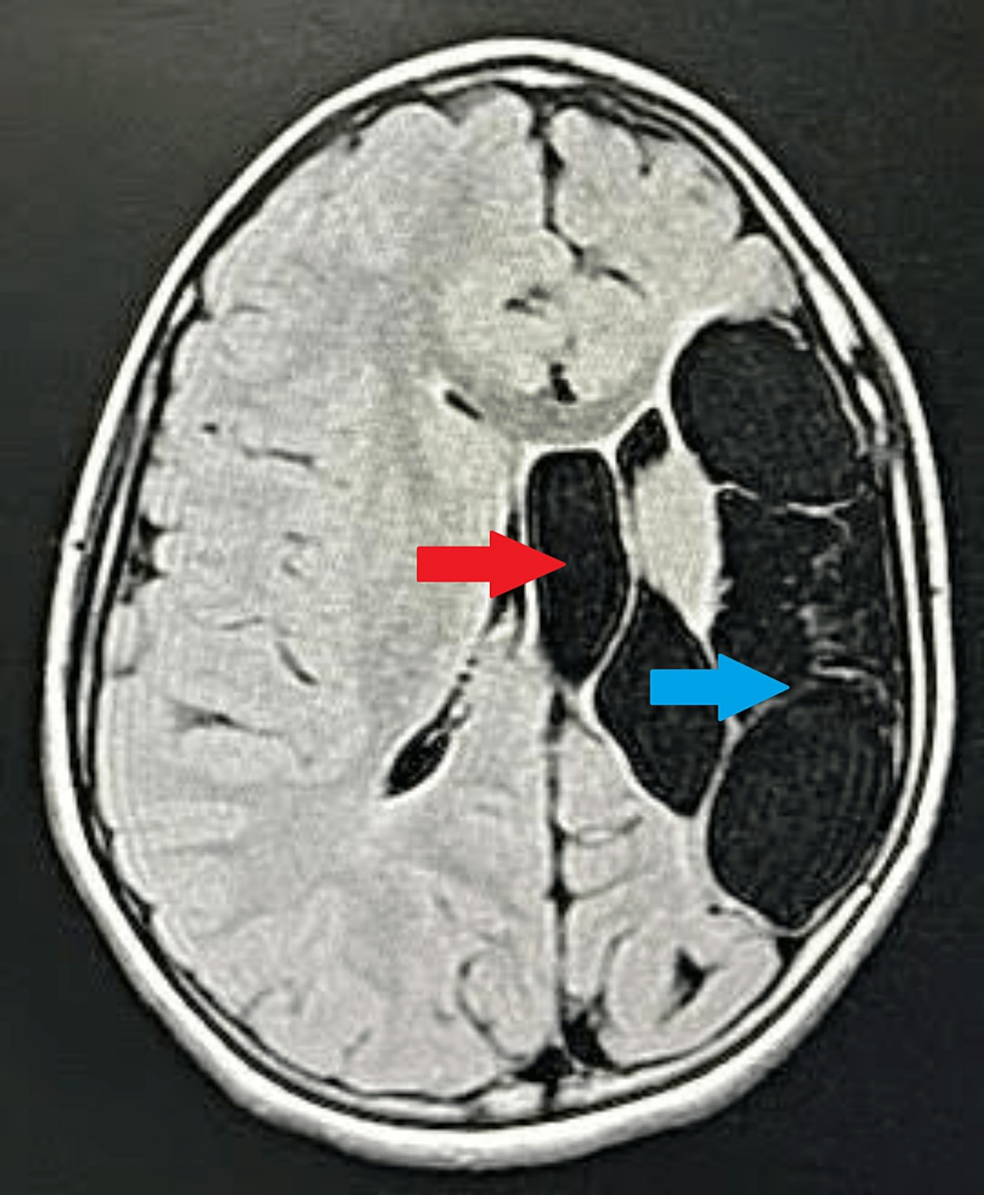
MRI brain axial FLAIR image Altered signal intensity is noted at the frontoparietal lobe on the left side with the loss of gray-white matter junction, encephalomalacia, and gliotic changes with dilatation of anterior horn and body of ipsilateral lateral ventricle appearing hypointense on FLAIR Red arrow: dilated left lateral ventricle. Blue arrow: altered signal intensity at the left frontoparietal lobe FLAIR: fluid-attenuated inversion recovery; MRI: magnetic resonance imaging

The patient was advised to undergo an MRI brain for further evaluation. On MRI brain axial sections, altered signal intensity appearing hypointense on T1 weighted imaging (T1WI) and fluid-attenuated inversion recovery (FLAIR) and hyperintense on T2 weighted imaging (T2WI) were noted at the frontoparietal lobe on the left side, with the loss of gray-white matter junction as well as encephalomalacia and gliotic changes with dilatation of anterior horn and body of ipsilateral lateral ventricle suggestive of significant left-sided partial cerebral hemiatrophy. Atrophy of the splenium of the corpus callosum, lentiform nucleus, basal ganglia, and internal capsule on the left side was noted (Figures 1-3).

**Figure 4:**
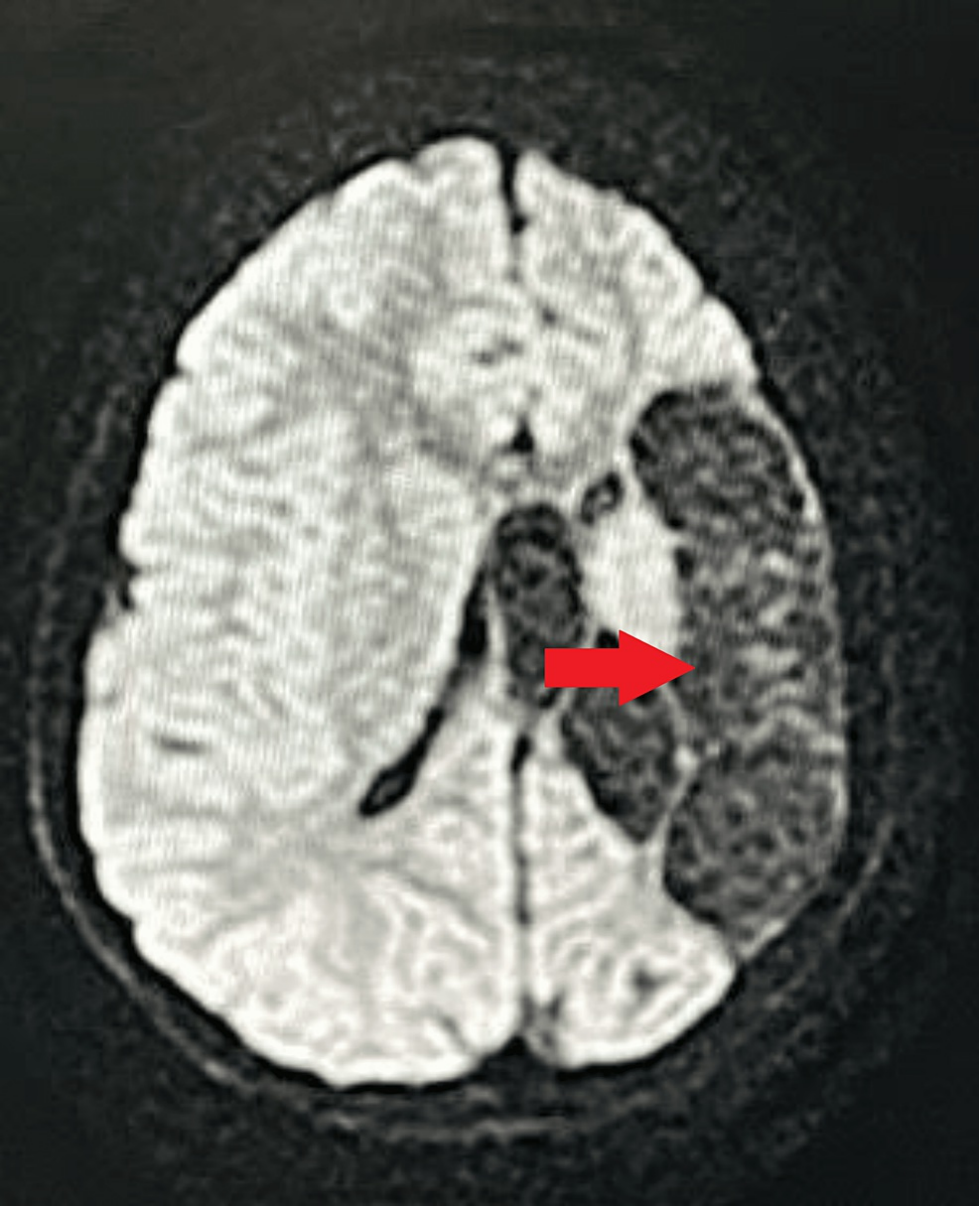
MRI brain axial DWI No evidence of restriction is noted at the frontoparietal lobe on the left side Red arrow: no evidence of restriction at the left frontoparietal lobe DWI: diffusion-weighted imaging; MRI: magnetic resonance imaging

**Figure 5:**
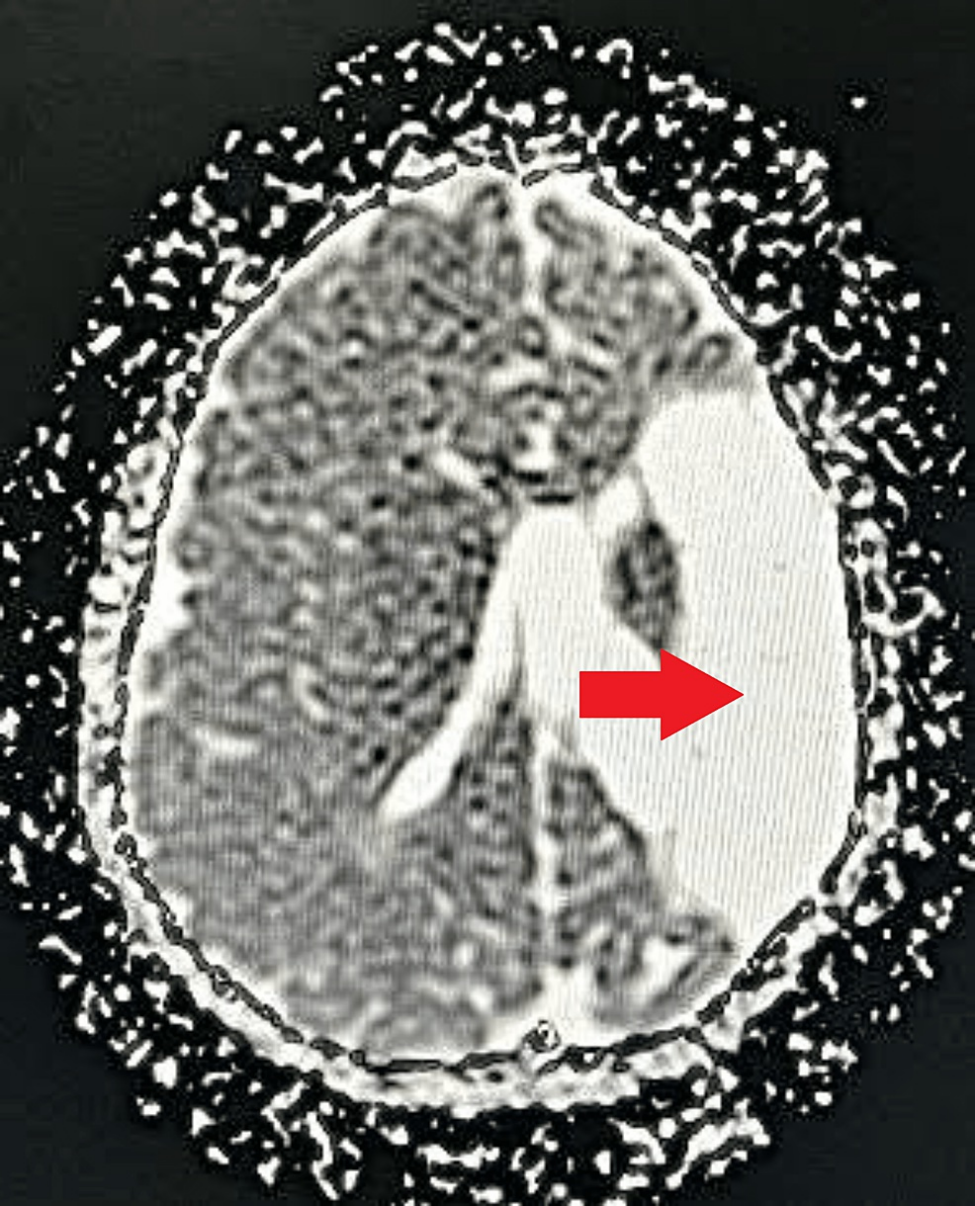
MRI brain axial ADC image Evidence of high ADC value is noted at the frontoparietal lobe on the left side Red arrow: high ADC value at the left frontoparietal lobe ADC: apparent diffusion coefficient; MRI: magnetic resonance imaging

No evidence of restriction was noted at the frontoparietal lobe on the left side on diffusion-weighted imaging (DWI) (Figure 4), corresponding to the high apparent diffusion coefficient (ADC) value (Figure 5).

**Figure 6:**
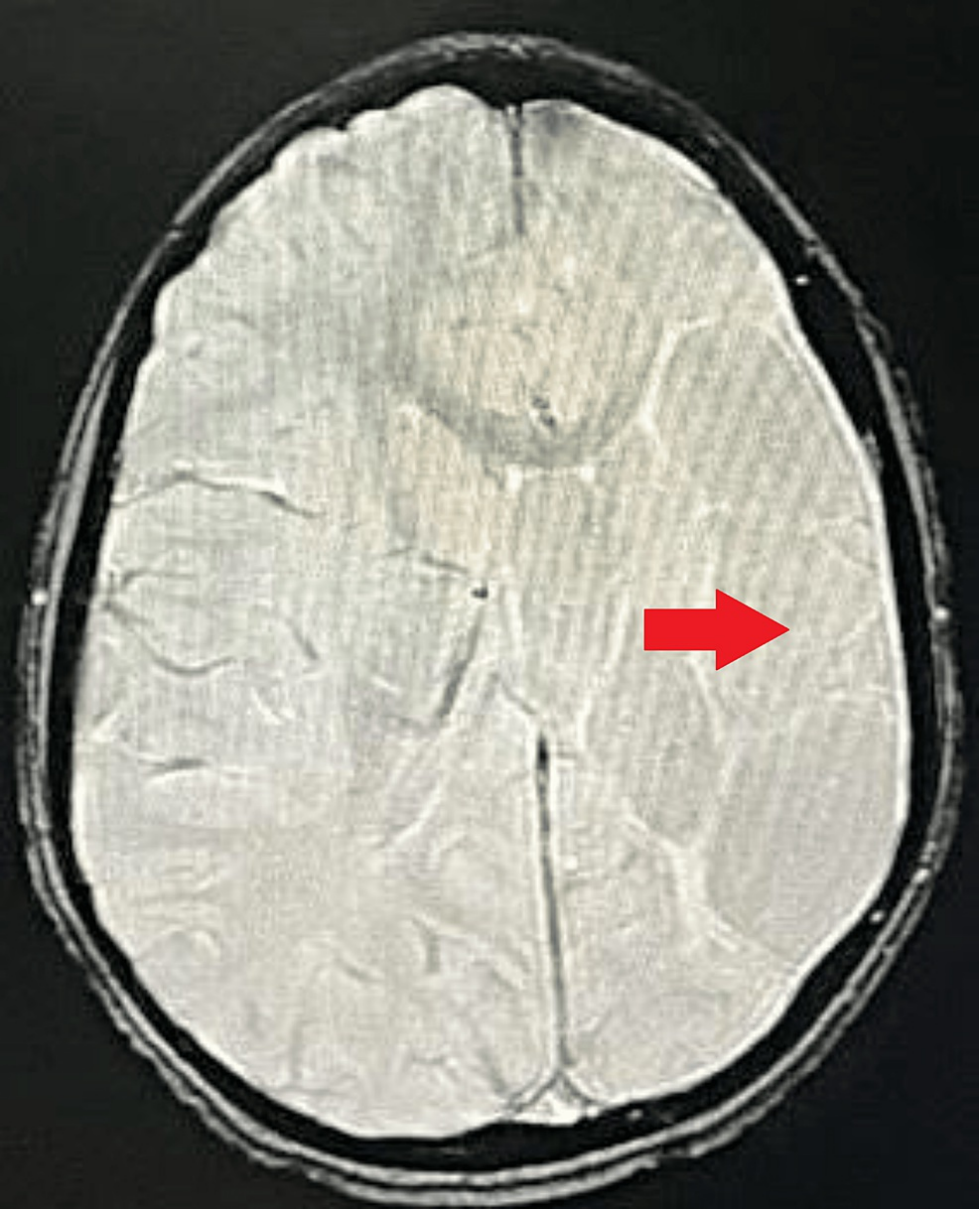
MRI brain axial SWAN image No evidence of blooming is noted Red arrow: no evidence of blooming at the left frontoparietal lobe SWAN: susceptibility-weighted angiography

No evidence of blooming was noted on star-weighted angiography (SWAN) (Figure 6).

Based on the above imaging features, a diagnosis of Rasmussen’s encephalitis was made. The patient was given IV immunoglobulin treatment for three months (details not available) when he was admitted to our hospital. Surgery was not planned. His condition subsequently improved, and he was advised to follow up after three months, but he was lost to follow-up.

## Discussion

Theodore Rasmussen first described Rasmussen’s encephalitis in 1958. Rasmussen’s encephalitis is a sporadic chronic inflammatory illness of the central nervous system that primarily affects children. The average age at presentation ranges from six to eight years. The effects are similar in both genders [1]. The neurological condition gradually deteriorates as a result of the cytotoxic T-cell reaction against the neuron, which causes the expression of MHC class I and apoptotic neuronal death [2].

Since imaging is a crucial tool for early diagnosis and tracking the development of the disease, the radiologist plays an active part in the diagnostic and treatment process. MRI of the brain exhibits unilateral enlargement of the CSF compartment, with the insular and peri-insular regions exhibiting the greatest accentuation, as well as the increased signal intensity in the cortical, subcortical, or both regions suggestive of Rasmussen’s encephalitis. The EEG displays subclinical ictal discharges, multifocal ictal discharges, focal sluggish activity, and unihemispherical attenuation of background activity and sleep spindles [3,4].

For certain cases, a brain biopsy is not necessary because the diagnosis can also be obtained without one. Therapy aims to reduce inflammation, regain functional ability, and manage seizures. Pharmacological, immunotherapeutic, surgical, and rehabilitative procedures are some of the different therapy modalities that can be used to accomplish these objectives [3]. The early institution of long-term immunotherapy to prevent functional decline is the recommended mode of therapy [2].

## Conclusions

Rasmussen’s encephalitis is an uncommon type of brain malformation characterized by chronic inflammatory changes of the unilateral cerebral hemisphere; it can be diagnosed accurately by MRI. Steroids, immunoglobulins, and surgery are the most effective treatment modalities for this condition.
